# The fungal natural product fusidic acid demonstrates potent activity against *Mycoplasma genitalium*

**DOI:** 10.1128/aac.01006-24

**Published:** 2024-08-29

**Authors:** Gwendolyn E. Wood, Jin Woo Lee, Thilini Peramuna, Karen L. Wendt, Caroline M. Kim, Laarni Kendra T. Aguila, Claire L. Calderon, Robert H. Cichewicz

**Affiliations:** 1 Department of Medicine, Division of Allergy and Infectious Diseases, University of Washington, Seattle, Washington, USA; 2 College of Pharmacy, Duksung Women’s University, Seoul, Republic of Korea; 3 Natural Products Discovery Group, Department of Chemistry and Biochemistry, Stephenson Life Sciences Research Center, Institute for Natural Products Applications and Research Technologies, University of Oklahoma, Norman, Oklahoma, USA; University Children's Hospital Münster, Münster, Germany

**Keywords:** *Mycoplasma genitalium*, fusidic acid, antimicrobial resistance, fungal natural products

## Abstract

Antimicrobial resistance is extremely common in *Mycoplasma genitalium*, a frequent cause of urethritis in men and cervicitis, vaginitis, and pelvic inflammatory disease in women. Treatment of *M. genitalium* infections is difficult due to intrinsic and acquired resistance to many antibiotic classes. We undertook a program to identify novel antimicrobials with activity against *M. genitalium* from fungal natural products. Extracts of *Ramularia coccinea* contained a molecule with potent activity that was subsequently identified as fusidic acid, a fusidane-type antibiotic that has been in clinical use for decades outside the United States. We found that minimum inhibitory concentrations of fusidic acid ranged from 0.31 to 4 µg/mL among 17 *M*. *genitalium* strains including laboratory-passaged and low-passage clinical isolates. Time-kill data indicate that bactericidal killing occurs when *M. genitalium* is exposed to ≥10 µg/mL for 48 h, comparing favorably to serum concentrations obtained from typical loading dose regimens. Resistance to fusidic acid was associated with mutations in *fusA* consistent with the known mechanism of action in which fusidic acid inhibits protein synthesis by binding to elongation factor G. Interestingly, no mutants resistant to >10 µg/mL fusidic acid were obtained and a resistant strain containing a F435Y mutation in FusA was impaired for growth *in vitro*. These data suggest that fusidic acid may be a promising option for the treatment of *M. genitalium* infections.

## INTRODUCTION

*Mycoplasma genitalium* is a slow-growing, atypical bacterium associated with reproductive tract disease including urethritis in men and vaginitis, cervicitis, and pelvic inflammatory disease in women (1). Recent surveillance data estimated that the overall prevalence of *M. genitalium* was 16.6% among individuals seeking care at sexual health clinics in six US cities (2).

Treatment of *M. genitalium* infections is becoming increasingly difficult. As *M. genitalium* lacks the targets of commonly used antimicrobials (e.g., peptidoglycan, outer membrane/LPS, and folic acid synthesis pathways), it is intrinsically resistant to these agents. An *rpoB* mutation common to all Mollicutes confers resistance to rifampin (3). Furthermore, the poor efficacy of doxycycline (30–40% effective) and increasing acquired resistance to macrolides (>60% of US strains) and fluoroquinolones (>10%) have resulted in the appearance of multidrug-resistant strains (4). Treatment of strains resistant to both azithromycin and moxifloxacin is challenging as few drugs with proven efficacy are available in the United States. For these reasons, *M. genitalium* was placed on the CDC Watch List of Antibiotic Resistance Threats in 2019.

To address the acute need for new treatments, we embarked on a collaborative effort to identify molecules with activity against *M. genitalium* within libraries of fungal natural products. Here, we report the identification of fusidic acid produced by *Ramularia coccinea*, its *in vitro* activity against multiple strains of *M. genitalium*, killing kinetics and mechanism of resistance. These data suggest that fusidic acid, an antibiotic used safely for decades outside the United States for other indications, may represent a promising option to treat drug-resistant *M. genitalium* infections.

## RESULTS

### Identification of fusidic acid in fungal extracts

**Fig 1:**
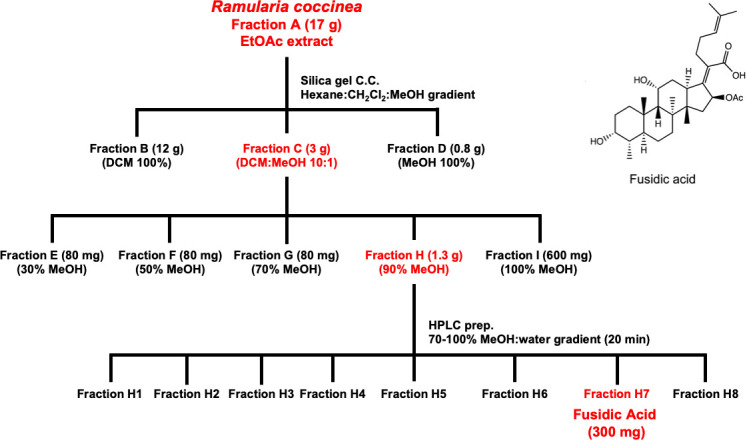
Bioassay guided fractionation of *Ramularia coccinea* extract to yield purified fusidic acid. The fractions with the highest activity are shown in red.

We screened approximately 4,200 extracts prepared from fungi that are part of the Natural Products Discovery Group library housed at the University of Oklahoma, a collection that contains fungi derived from diverse ecological niches across the United States. Initial library screening demonstrated that a fungus identified by ITS sequencing as *R. coccinea* produced a substance with activity against *M. genitalium*. The minimum inhibitory concentration (MIC) of the crude extract was 31 µg/mL as determined in microbroth dilution assays. Cytotoxicity assays were performed against Vero cells to assess selectivity for *M. genitalium*. Vero cells were exposed to crude extracts for 48 h then cytotoxicity was measured using an Alamar blue reduction assay. We detected no cytotoxicity with crude *R. coccinea* extract as high as 775 µg/mL (25× MIC). Fresh crude extracts from scale-up cultures were also active against *M. genitalium* (MIC 2 µg/mL) thus confirming that the active molecule is consistently produced by this fungus. Through a process of bioassay-guided fractionation (Fig. 1), we identified a fraction containing a single purified compound. Analysis of spectroscopic data determined that the compound was fusidic acid (Fig. S1 and S2), a fusidane triterpene-based antibiotic first identified in the 1960s as a natural product produced by *Fusarium coccineum* (5). Fusidic acid is approved for clinical use outside the United States (>20 countries) in oral, intravenous, or topical forms to treat various types of *Staphylococcus aureus* infections (e.g., wound infections, pneumonia, osteomyelitis, and septicemia) (6). Helvolic acid—another fusidane-type molecule obtained from our NPDG pure compound library—was also active against *M. genitalium* with complete inhibition of growth by ≤50 µM. As helvolic acid is not used clinically, its activity against *M. genitalium* was not investigated further.

### Susceptibility of *M. genitalium* strains to fusidic acid

**Fig 2:**
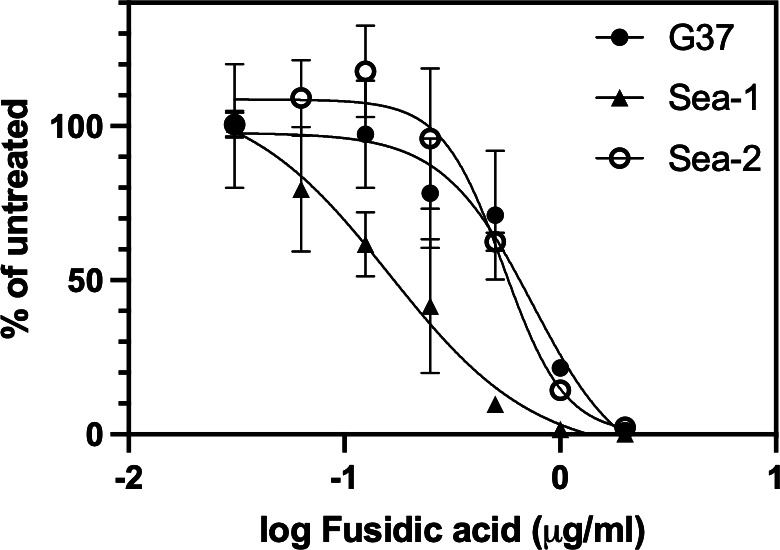
Growth inhibition dose response of *M. genitalium* strains G37, Sea-1, and Sea-2 to fusidic acid. The *y*-axis shows the mean number genomes detected in triplicate qPCR measurements as a percentage of untreated *M. genitalium*. Errors bars show standard deviation of triplicate drug-treated wells. The experiment was repeated two times with similar results.

The fusidic acid MIC was determined for eight *M. genitalium* strains, including the G37 type strain and other laboratory passaged, broth-adapted strains using commercially available fusidic acid. MICs, determined by assessing color change in microbroth dilution assays, ranged from 0.63 to 2.5 µg/mL MICs (Table 1). To precisely measure inhibition, and confirm dose response, we quantified the growth of three strains of *M. genitalium* in these microbroth dilution assays by qPCR. The IC_50_ for these strains was similar: 0.59 ± 0.17 for G37, 0.20 ± 0.08 for Sea-1, and 0.55 ± 0.07 for Sea-2 (Fig. 2).

**TABLE 1:** *In vitro* susceptibility of *M. genitalium* strains to fusidic acid^*f*^

Strain designation	Strain type*^a^*, year of isolation, location	MIC (µg/mL)			
		Fusidic acid	Doxycycline	Moxifloxacin	Azithromycin
Microbroth dilution*^b^*					
G37	J-1, 1980, United Kingdom	1.25	0.25	0.125	0.002
Sea-1	J-39, 1998, Seattle, WA, USA	0.63	0.004c	ND	0.002
Sea-2	J-6, 1998, Seattle, USA	2.5	0.004	ND	0.002
M30	J-2, 1980, United Kingdom	2.5	0.5	0.125	0.008
TW60	ND, 2000, San Antonio TX, USA	1.25	ND	ND	ND
M2282	J-5, 1991, Denmark	0.31	0.5	0.25	<0.002
M2300	J-20, 1991, Denmark	1.25	0.125	0.125	<0.002
M2341	J-2, 1991, Denmark	0.63	0.04	ND	ND
Vero cell coculture*^c^*					
MEGA 216	J-39, 2008, Seattle, WA, USA	<2	2	ND	>8 A2058C*^d^*
MEGA 552	J-6, 2008, Seattle, WA, USA	<4	1	ND	>8 A2058G
MEGA 601	J-2, 2008, Seattle, WA, USA	2	0.25	ND	0.004
MEGA 1082	GB-6, 2009, Seattle, WA, USA	4	0.25	>1 G248T (Ser83I)*^e^*	>8 A2058G
MEGA 1202	43ND, 2009, Seattle, WA, USA	4	0.5	ND	>8 A2059G
MEGA 1256	GB-2, 2009, Seattle, WA, USA	<2	0.25	ND	>8 A2058G
MEGA 1272	J-51, 2009, Seattle, WA, USA	<2	0.25	ND	>8 A2059G
MEGA 1568	ND, 2010, Seattle, WA, USA	<2	0.5	ND	<0.001
MEGA 1606	ND, 2010, Seattle, WA, USA	4	1	ND	0.002

^*a*^ *mgpB* strain type determined as previously described (7, 8).

^*b*^ MIC defined as lowest concentration with no color change.

^*c*^ MIC is the concentration inhibiting growth by ≥99% as compared to untreated *M. genitalium* determined by qPCR.

^*d*^ Macrolide resistance mutation in 23S rRNA gene (7).

^*e*^ Quinolone resistance mutation in the *parC* gene (amino acid change).

^*f*^ ND, not determined.

To determine if fusidic acid susceptibility is a general characteristic of *M. genitalium* strains, we determined MICs for nine low-passage clinical isolates that have not been adapted to axenic culture and are dependent on Vero coculture for growth. These strains were isolated from men with non-gonococcal urethritis who were enrolled in a trial comparing azithromycin and doxycycline for treatment of *M. genitalium* infection conducted from 2007 to 2011 (7), and represent a variety of *mgpB* strain types and azithromycin and moxifloxacin resistance mutations (9). Fusidic acid MICs for these clinical isolates ranged from <2 to 4 µg/mL. Considering all *M. genitalium* strains tested the MIC_50_ was 2 µg/mL and the MIC_90_ was 4 µg/mL.

### Bactericidal activity of fusidic acid

**Fig 3:**
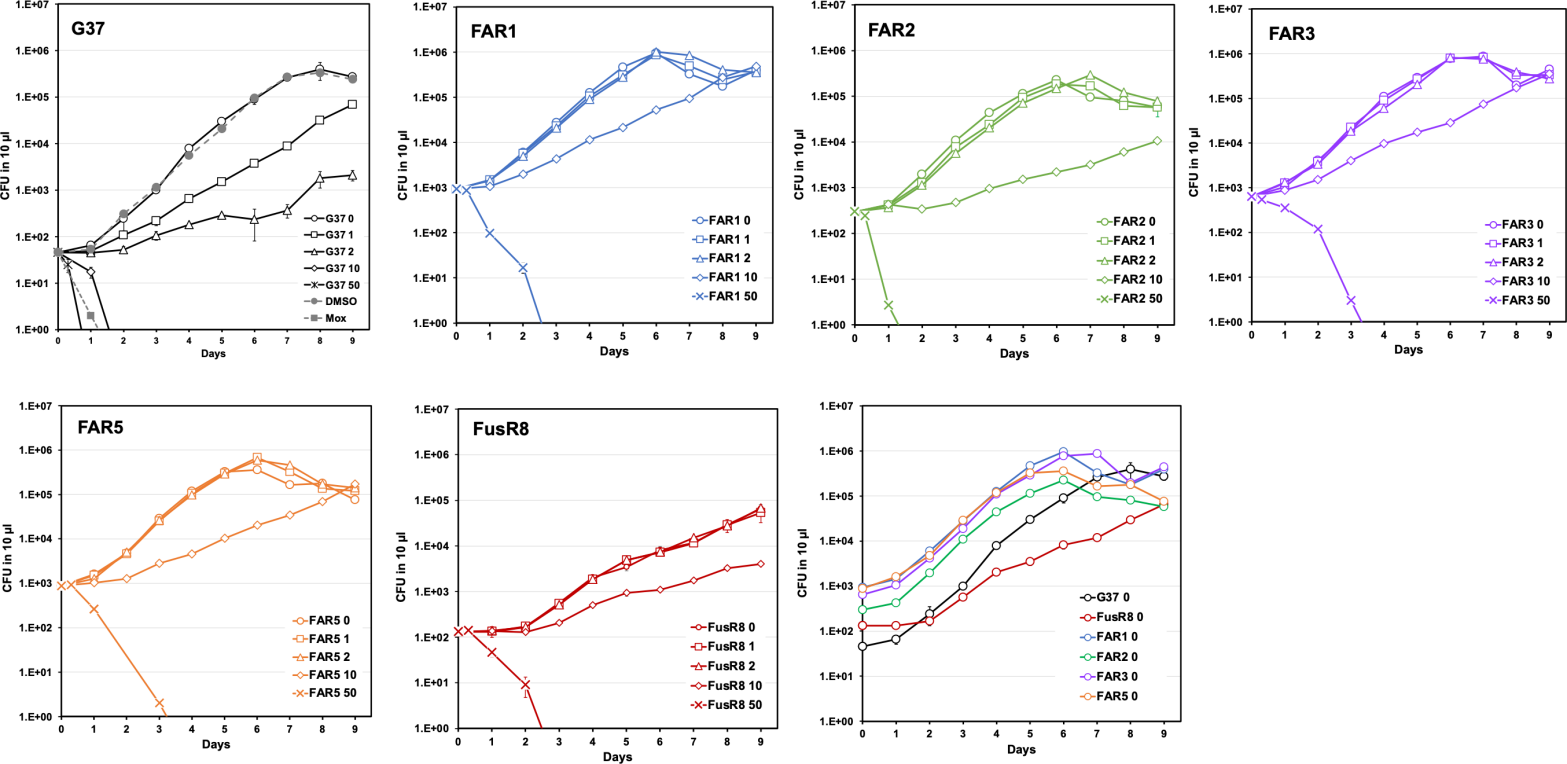
Time-kill experiments. Wild-type and fusidic acid-resistant *M. genitalium* were treated with various concentrations of fusidic acid over time. Curves show the average colony-forming units per 10 µL aliquot spotted in triplicate on SP-4 agar plates. Error bars indicate standard deviation. Dimethyl sulfoxide (DMSO, solvent control) was added at 0.5% corresponding to the highest drug concentration. Moxifloxacin (Mox, positive control) was used at 0.25 µg/mL. The last panel shows the growth of each strain in the absence of fusidic acid. Results of a typical experiment repeated two times are shown.

Time-killing kinetics were investigated by exposing *M. genitalium* strain G37 to fusidic acid concentrations ranging from 0 to 50 µg/mL in SP-4 broth cultures. Aliquots were removed at intervals (0 and 8 h, then daily for 9 days) and then dilution plated on SP-4 agar plates in triplicate. As shown in Fig. 3 (upper left), fusidic acid at 1 and 2 µg/mL inhibited the growth of wild-type strain G37 by 90–99% as compared to untreated or solvent control-treated cultures. Fusidic acid at 10 and 50 µg/mL was bactericidal and killed >99.9% of *M. genitalium* in 48 or 24 h, respectively.

### Resistance to fusidic acid

To investigate fusidic acid resistance potential in *M. genitalium,* we determined the resistance rate by plating *M. genitalium* strain G37 onto SP-4 agar plates containing fusidic acid at 10, 25, or 50 µg/mL or on SP-4 agar without fusidic acid to quantify the inoculum. Colonies were visible after 2 weeks of incubation on 10 µg/mL fusidic acid, but no colonies appeared on 25 or 50 µg/mL fusidic acid. When compared to the inoculum, the resistance rate was calculated as ~5 × 10^−7^ on 10 µg/mL and <3 × 10^−7^ on 25 and 50 µg/mL. The colonies growing on 10 µg/mL had an atypical morphology (flat colonies rather than the “fried egg” morphology characteristic of *M. genitalium*). All eight colonies grew when subcultured to plain SP-4 broth, but fewer than half grew in 10 µg/mL fusidic acid suggesting that not all clones were truly resistant. Four clones (named FAR1, FAR2, FAR3, and FAR5) that grew at 10 µg/mL were chosen for further analysis. In a complementary strategy, we isolated fusidic acid-resistant mutants by serial passage of strain G37 in increasing concentrations of fusidic acid. This approach yielded a culture that grew slowly in 3 µg/mL fusidic acid. We obtained single colonies after filtering through 0.45 µm and then characterized the resulting clone, FusR8, as described below.

### Characterization of fusidic acid-resistant *M. genitalium*

Using microbroth dilution assays, we determined that the MIC for each of the five fusidic acid-resistant mutants was 6.3 µg/mL, fivefold higher than wild-type strain G37. Growth curve and time-kill experiments were performed with each resistant mutant (Fig. 3). As expected, growth of these resistant strains was unaffected by fusidic acid at 1 and 2 µg/mL. Growth was inhibited 94–99% by 10 µg/mL and >99.9% killing was observed after 48–96 h in 50 µg/mL. Interestingly, growth of one mutant was slower than the parent strain in plain SP-4 with doubling times of 13.1 ± 1.82 and 27.8 ± 5.47 h for G37 and FusR8, respectively (*P* = 0.006, Student’s one-tailed *t* test for independent samples). The growth rate of fusidic acid-resistant mutants FAR1, FAR2, FAR3, and FAR5 did not differ significantly from wild-type G37 with doubling times ranging from 11.5 to 12.5 h.

We tested whether fusidic acid resistance affected the susceptibility of *M. genitalium* to doxycycline and moxifloxacin in the resistant mutants. All of the fusidic acid-resistant mutants had MICs for doxycycline and moxifloxacin identical to the parent G37 strain (0.25 and 0.125 µg/mL, respectively). Furthermore, clinical isolates resistant to azithromycin and/or moxifloxacin had low fusidic acid MICs that were similar to strains that are susceptible to these antibiotics (Table 1) thus supporting that resistance to macrolides and fluoroquinolones does not affect susceptibility to fusidic acid.

### Identification of fusidic acid resistance-associated mutations

**Fig 4:**
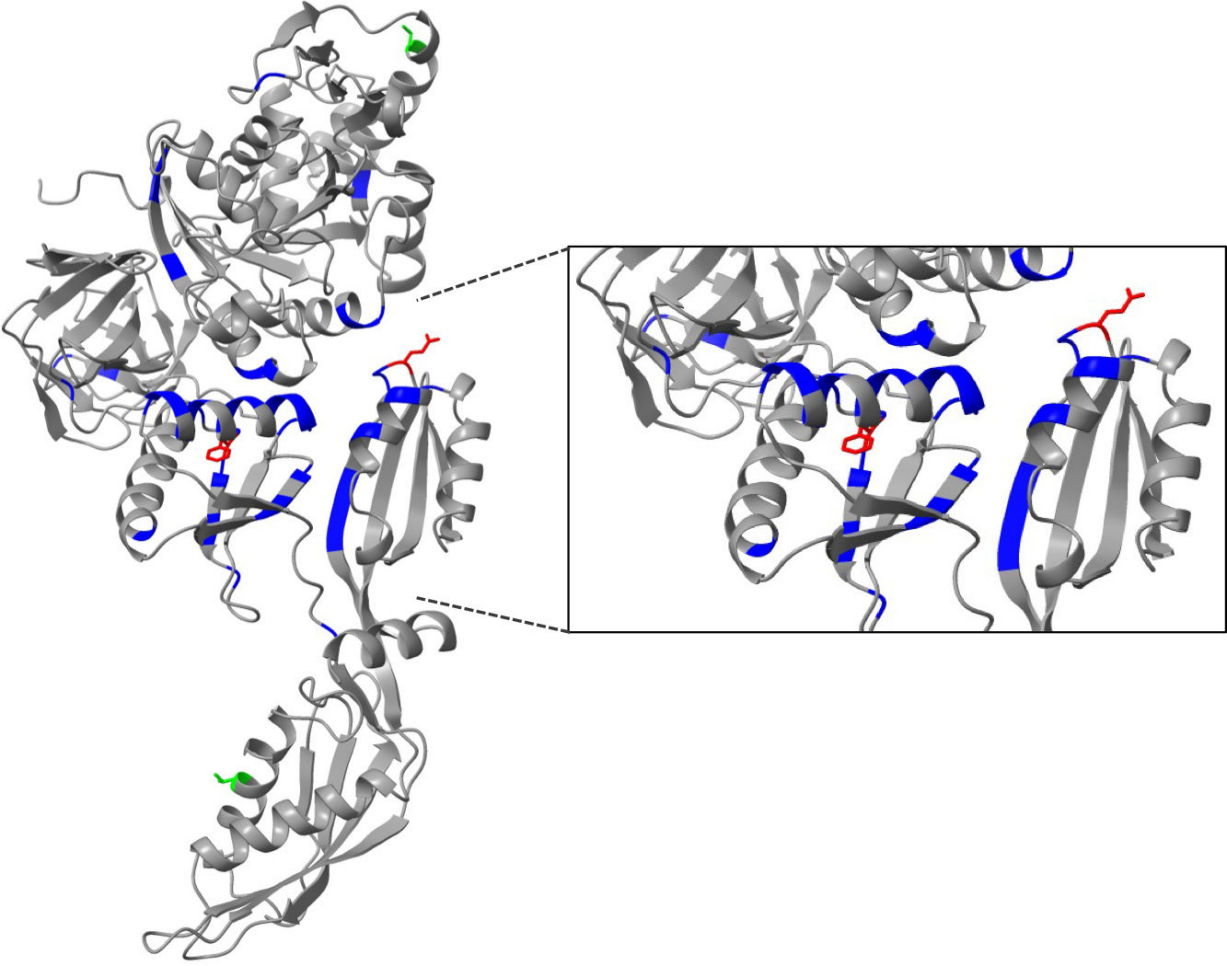
Structure of *M. genitalium* FusA predicted by AlphaFold (P47335). Close-up shows fusidic acid binding pocket. The locations of amino acids associated with fusidic acid resistance are shown in blue (*S. aureus*) and red (*M. genitalium*). Two variant residues identified among *M. genitalium* strains that are unlikely to affect fusidic acid susceptibility are shown in lime green.

Whole-genome sequencing was performed on all five fusidic acid-resistant clones to identify resistance associated mutations and infer the target of fusidic acid in *M. genitalium*. We identified a single base change in the *MG_089* gene, encoding FusA, also known as elongation factor G (EF-G), in all five fusidic acid-resistant mutants that were not present in the wild-type G37 parent strain maintained in our laboratory [sequenced previously (10)]. Mutants FAR1, FAR3, and FAR5 each acquired an A to G point mutation at base pair 116,786 (bp 1,979 of *fusA*) predicting a Q660R mutation in FusA. Mutant FAR2 contained a C to A mutation at bp 116,785 (bp 1,978 of *fusA*) encoding a Q660K mutation. A single T to A point mutation at base pair 116,111 (bp 1,304 of *fusA*) occurred in the FusR8 mutant predicting an F435Y mutation in FusA. The absence of other mutations in FusR8 suggested that the F435Y mutation also affected the growth of this strain. The presence of mutations in *fusA* is consistent with the known mechanism in which fusidic acid binds to FusA to inhibit translation. The *S. aureus* FusA has been co-crystallized with fusidic acid (11). Given the similarity of the structure of *M. genitalium* FusA predicted by AlphaFold (PDB P47335) to *S. aureus* FusA, the F435Y mutation is likely to affect the fusidic acid binding pocket, whereas Q660L/R may affect the interaction of EF-G with the ribosome (11). Figure 4 shows the location of the *M. genitalium* and *S. aureus* resistance-associated mutations (6, 11) (in red and blue, respectively) mapped onto the predicted *M. genitalium* FusA structure. These data provide further evidence that fusidic acid prevents the growth of *M. genitalium* by binding to FusA and inhibiting protein synthesis (12).

### Fusidic acid resistance-associated mutations in *Mycoplasma* spp.

To determine if fusidic acid resistance mutations are present among *M. genitalium* strains, we aligned the G37 FusA protein sequence with that of 23 fully genome-sequenced strains (13). FusA is highly conserved among these strains with variability at only two residues: V or A at amino acid 204 and T or A at amino acid 540. These amino acids are distant from the fusidic acid binding pocket (Fig. 4, lime green) and neither of these residues is implicated in fusidic acid resistance in *S. aureus* (6, 11). Furthermore, no *M. genitalium* strains had mutations in F435 or Q660. Taken together, these results suggest that most *M. genitalium* strains express a fusidic acid susceptible FusA. In addition, F435 and Q660 are conserved in FusA among more than 20 *Mycoplasma* species. F435 is conserved among four *M. fermentans* sequenced strains (PG18, JER, MF-I1, and M64), but Q660 is conserved in only one strain (PG18) and three strains (JER, MF-I1, and M64) instead have A660. Interestingly, *M. fermentans* fusidic acid MICs ranged from 2.5 to 25 µg/mL with an MIC_50_ of 10 µg/mL and MIC_90_ of 25 µg/mL suggesting that most strains are resistant (14). It is tempting to speculate that the Q660A mutation confers fusidic acid resistance in these *M. fermentans* strains.

## DISCUSSION

Antimicrobial resistance complicates the treatment of most *M. genitalium* infections. This unique bacterium lacks a cell wall and outer membrane rendering antibiotics targeting these structures, such as b-lactams and colistins, respectively, entirely ineffective. Additionally, a mutation in *rpoB* common to all Mollicutes imparts resistance to rifampins, and the absence of folic acid synthesis pathways in these organisms makes treatment with sulfonamides and trimethoprim futile. Natural product libraries are invaluable for identifying agents effective against *M. genitalium*, including novel molecules like xanthoquinodins, N-hydroxypyridones, and tetramic acids [(15, 16) and Peramuna et al., submitted]. Additionally, they have helped uncover new activities in previously known agents, including nitroimidazoles (10) and fusidic acid, as outlined herein. We demonstrated that fusidic acid has potent activity (MIC_90_ = 4 µg/mL) against a variety of laboratory-passaged and low-passage clinical isolates of *M. genitalium*. Bactericidal killing was observed when *M. genitalium* was exposed to ≥10 µg/mL of fusidic acid, well below plasma concentrations resulting from typical treatment regimens (12). Mutations in *M. genitalium fusA* confer resistance to fusidic acid consistent with direct interaction of this drug with EF-G and its known mechanism of action. Although we obtained resistant *M. genitalium in vitro*, at least one strain had a reduced growth rate suggesting that some mutations could confer a competitive disadvantage. Fusidic acid may prove useful as an alternative treatment for multidrug-resistant *M. genitalium* or in individuals for whom current front-line agents are contraindicated.

Fusidic acid, a fusidane triterpene-based antibiotic, was first identified in the 1960s as a natural product produced by *Fusarium coccineum* that inhibits primarily Gram-positive organisms. Approved for clinical use outside the United States (>20 countries) for decades, fusidic acid is available in oral, intravenous, or topical forms for indications such as methicillin-resistant *S. aureus* infection. Fusidic acid binds to EF-G, a component of the ribosome that catalyzes the translocation of the growing peptide from the A to P site. When fusidic acid binds EF-G, the complex is trapped in the A site and protein synthesis is stalled. Dozens of fusidic acid analogs have been tested including synthetic (5, 17) and naturally occurring (cephalosporin P and helvolic acid) molecules. None are more potent than fusidic acid against susceptible Gram-positive organisms, but one analog has improved activity against fusidic acid-resistant *S. aureus* both *in vitro* and in a mouse thigh infection model (18).

The mechanisms of fusidic acid resistance have been extensively studied, particularly in *S. aureus* as fusidic acid is indicated for the treatment of local and systemic infections (12). In staphylococci, fusidic acid resistance (MIC >1 µg/mL as defined by the European Committee on Antimicrobial Susceptibility Testing) arises from spontaneous mutations in *fusA* or *rplF* with different mutations conferring different levels of resistance. For example, *S. aureus* FusA P406L mutants are resistant to 8 µg/mL, H457Y to 64 µg/mL, and L461K to >256 µg/mL as compared to 0.032 µg/mL for the parent susceptible strain (19). Some mutations conferring high-level resistance (e.g., *S. aureus* FusA F88L MIC >64 µg/mL) also affect growth rate (20), similar to the *M. genitalium* FusA F435Y mutation identified in this study. However, secondary mutations (e.g., M16I) can restore fitness in *S. aureus* without reducing MIC. Because of the high rate of spontaneous resistance, a second antibiotic is recommended (e.g., rifampin) to reduce selection of resistant *S. aureus* and improve treatment outcomes (12). However, recent data suggest that co-administration of rifampin lowers plasma concentrations of fusidic acid potentially reducing clinical efficacy and increasing the opportunity for resistance development (21). As noted above, all Mollicutes are resistant to rifampin so this strategy would be ineffective in reducing fusidic acid resistance development in *M. genitalium*.

In *S. aureus*, the frequency of spontaneous resistance *in vitro* decreases with higher fusidic acid concentrations: 10^−6^ at 2× MIC versus 10^−8^ at 16× MIC (12). We observed a similar phenomenon, where fewer resistant *M. genitalium* colonies emerged at higher fusidic acid concentrations, and no clones capable of consistent growth in concentrations greater than 25 µg/mL were obtained. Future experiments will assess whether high-level resistance can be selected during long-term passage in low concentrations of fusidic acid, and whether second-site mutations can restore normal growth in the F435Y mutant.

Although more than 30 resistance mutations in *fusA* have been described in *S. aureus* during *in vitro* selection and in clinical isolates, fusidic acid resistance in staphylococci more commonly develops via horizontal acquisition of the *fusB*, *fusC*, *fusD*, or *fusF* genes encoding EF-G protection proteins (6, 22). These small proteins, each under 25 kDa, interact with EF-G when fusidic acid is bound inducing a conformational shift, which releases EF-G from the stalled ribosome complex allowing translation to resume. Acquisition of resistance genes via horizontal transfer has not been demonstrated in *M. genitalium* clinical isolates although a mechanism for low-frequency horizontal gene transfer *in vitro* has been described (23). The non-canonical genetic code used by *M. genitalium* in which the typical TGA stop codon encodes tryptophan (24) may hinder gene acquisition from other bacterial species.

Safety and pharmacokinetics of fusidic acid have been well documented. Single-dose fusidic acid results in high plasma concentrations ranging from 33 µg/mL for 550 mg to 93 µg/mL for 1650 mg (25). When a loading dose regimen is used (e.g., 1650 mg bid, then 825 mg bid) mean trough plasma concentrations reach 146 µg/mL at 24 h rising to 204 µg/mL after 8 days. Importantly, these high doses were well tolerated and effective in a US phase 2 trial for acute bacterial skin and skin structure infections (25, 26). Compared to our *in vitro* killing data, these pharmacokinetic data suggest that cure of both fusidic acid susceptible and resistant strains of *M. genitalium* may be achieved with high dose, short duration treatment.

Fusidic acid activity is affected by pH which may be relevant to treatment of *M. genitalium* infections. Acidic growth conditions (pH 5–5.5) reduce fusidic acid MICs for *S. aureus*, and enhance the accumulation of the drug within the bacterial cell approximately fourfold as compared to pH 7 (27). Fusidic acid is highly protein bound in neutral pH (>95%); however, in acid pH protein binding is reduced thereby increasing the proportion of free drug and reducing the MIC (28). Fusidic acid accumulates in macrophages in neutral pH where it can kill intracellular bacteria, and intracellular concentrations are further increased in low pH (27, 28), an ability that may enhance clearance of intracellular *M. genitalium* (29–31). These phenomena may suggest that the low pH of the vagina, or inflamed microenvironments in other tissues, would increase fusidic acid potency against *M. genitalium*. Fitzgerald et al. found that a strain of *Enterococcus faecalis* resistant to fusidic acid due to a FusA C316A mutation developed compensatory mutations in *fusA* during *in vitro* passage in low pH (4.8) medium. Interestingly, the second-site mutations selected in low pH also restored fusidic acid susceptibility (32). The authors suggest that the growth in low pH could select against certain fusidic acid resistance alleles.

Other properties of fusidic acid may enhance its activity *in vivo*. Fusidic acid has anti-inflammatory activity as demonstrated in a mouse ear edema model (33), which may improve symptoms of infection, similar to azithromycin (34). The lipophilicity and large size of fusidic acid impede its passage through the Gram-negative outer membrane, rendering fusidic acid ineffective against Enterobacterales. This suggests that fusidic acid may have a lesser effect on the microbiome compared to other broad-spectrum antibiotics (6). Finally, the chemical scaffold and mechanism of action of fusidic acid differ from other antimicrobials, so cross-resistance between fusidic acid and other antibiotics does not occur (12).

Importantly, fusidic acid has *in vitro* activity against *Neisseria gonorrhoeae* and *Chlamydia trachomatis* (35), sexually transmitted bacterial pathogens with similar symptomology. In addition, 10–25% of patients with *M. genitalium* infections are also co-infected with one or both of these pathogens (1). A drug that treats all three pathogens would be invaluable, especially in resource poor settings where sexually transmitted infections are managed syndromically.

## MATERIALS AND METHODS

### Strains, media, and antibiotics

*M. genitalium* strains used in this study comprised strains capable of axenic growth including the G37 type strain (36), M30, M2282, M2300, and M2341 (37), and Sea-1 and Sea-2 (38). In addition, nine low-passage clinical strains cultured from men with urethritis were chosen as representatives of a variety of strain types with known resistance profiles to azithromycin, doxycycline, and moxifloxacin (7). Axenic strains were grown in SP-4 (39) and clinical isolates were grown in Vero cell co-cultures in EMEM (Corning Life Sciences) supplemented with 10% fetal bovine serum (FBS; RND Systems), 6% yeast dialysate, and 25 mM HEPES, pH 7.2 as previously described (7). Antibiotics for susceptibility testing were purchased from Sigma and dissolved in water (moxifloxacin, doxycycline), DMSO (fusidic acid), or 95% ethanol (azithromycin) and stored in aliquots at −20°C. Helvolic acid was obtained from the NPDG collection maintained at the University of Oklahoma.

### Fungal isolates and fermentation

The *Ramularia* sp. isolate (TX10278 TV8-5) was obtained from soil sample collected from a garden near Texarkana, TX, USA. The fungus was identified by collecting mycelium and subjecting the samples to homogenization in TE buffer (10 mM EDTA HCl, 0.1 mM EDTA, pH 8.0) with zirconium oxide beads in a Bullet Blender (MidSci #BBY24M). The DNA was subsequently collected, and the ribosomal internal transcribed spacer region and the 5.8S rRNA genes were amplified by PCR for sequencing. The resulting sequence data were compared to fungal sequences contained in GenBank, which led to 100% identity matches to isolates described as *R. coccinea* (isolate from TX, USA).

To prepare the isolates for chemical studies, fungi were recovered from cryogenic storage (stored in a vial at −80°C as mycelium with 20% aqueous glycerol). Following recovery on Czapek agar plates (30 g sucrose, 2 g NaNO_3_, 1 g K_2_HPO_4_, 0.5 g MgSO_4_⋅7 H_2_O, 0.5 g KCl, 0.01 g FeSO_4_⋅7 H_2_O, 0.05 g chloramphenicol, and 1 L DI H_2_O), lawns of fungal mycelium were aseptically cut into small pieces (~1 cm^2^) for use as the scale-up culture inoculum. Scale-up cultures were carried out by charging mycobags (Unicorn Bags, Plano, TX, USA) with monolayers of Cheerios breakfast cereal supplemented with a 0.3% sucrose solution and 0.005% chloramphenicol. The pieces of mycelium were aseptically added to three mycobags and the cultures were grown at room temperature for 4 weeks.

### Extraction, purification, and identification of fusidic acid

*R. coccinea* cultured on Cheerios cereal in the three mycobags was extracted with 2 L ethyl acetate (×3) at room temperature, the organic solvent layers were recovered, and the solvent was removed under vacuum. The crude EtOAc (fraction A, 17 g) was subjected to silica gel vacuum column chromatography with elution performed using dichloromethane (fraction B), dichloromethane-MeOH (10:1) (fraction C), and MeOH (fraction D). Fraction C (3 g) was also further fractionated by HP20ss gel vacuum column chromatography into five samples: fractions E (30% MeOH), F (50% MeOH), G (70% MeOH), H (90% MeOH), and I (100% MeOH). Fraction H (1.3 g) was further subjected to preparative HPLC (C_18_, gradient elution with 70–100% MeOH in H_2_O over 20 min using a 10 mL/min flow rate) to afford eight subfractions (H1–H8). Among these subfractions, H-7 was identified as fusidic acid (300 mg) by comparing the physicochemical and spectroscopic data with published values (Supporting Information Fig. S1 and S2) (40).

### Microbroth dilution assays

Minimum inhibitory concentrations of axenic strains were determined in microbroth dilution assays as previously described (10). Briefly, *M. genitalium* cultures were grown to late log phase, scraped, passed through a 0.45-µm filter to remove aggregates, then diluted to 10^5^ colony-forming units per mL. Dilutions of fusidic acid or doxycycline (comparator) were prepared in 0.1 mL in 96-well plates then 0.1 mL of the inoculum was added to each well. Plates were incubated at 37°C with 5% CO_2_ in a humidified atmosphere until wells containing no drug turned from red to yellow (indicating fermentation of glucose and late log phase growth). The MIC was identified as the lowest concentration of drug-inhibiting growth (no color change). As growth rates varied between axenic strains, incubation times ranged from 6 to 14 days.

### Time-kill experiments

Time-kill experiments were performed as previously described (10, 41). Adherent, log phase *M. genitalium* strain G37 was scraped off plastic petri dishes into the culture supernatant, filtered through 0.45 µm, and then diluted to 10^4^–10^5^ CFU per mL in 3 mL SP-4 broth containing DMSO (0.5%, solvent control corresponding to the highest drug concentration), or 1, 2, 10, or 50 µg/mL of fusidic acid. Immediately after inoculation and at intervals during 7–10 days incubation, the tubes were vortexed, 10-fold serial dilutions were prepared, and 10 µL aliquots were spotted onto SP-4 agar plates in triplicate. Colonies were counted under 40× magnification after 2–3 weeks of incubation. Control cultures treated 1, 0.5, 0.25, or 0.125 µg/mL of moxifloxacin have been previously reported (10). Doubling times were calculated using an online tool (https://www.omnicalculator.com/biology/bacteria-growth).

### Antibiotic susceptibility testing of Vero cell-dependent clinical isolates

To determine the MIC for *M. genitalium* clinical isolates, we based our protocol on the methods of Hamasuna et al. (7, 37). Vero cells (1 × 10^5^ cells) were cultured for 1 day in 25 cm^2^ tissue culture flasks with Eagles minimal essential medium (EMEM; Corning) supplemented with 10% FBS (RND Systems), 25 mM HEPES, and penicillin (100 U/mL). Fresh media (4 mL, EMEM supplemented with 10% FBS, 25 mM HEPES, 100 U/mL penicillin, 30 µg/mL colistin, and 6% yeast dialysate) was added containing serial twofold dilutions of fusidic acid or control antibiotics. The flasks were incubated for 28 days at 37°C in 5% CO_2_ and aliquots of culture supernatants were collected weekly to detect growth by *M. genitalium*-specific quantitative PCR. Each aliquot was quantified in triplicate qPCR reactions to verify growth (>100-fold increase in genomes/mL), identify the time point representing late log phase growth (generally 21 or 28 days of incubation), and determine MIC. MICs were defined as the minimum concentration of antibiotic that inhibited growth by ≥99% compared to the growth of each strain in control flasks containing no antibiotic.

### Quantification of *M. genitalium* growth by qPCR

To obtain precise measurements of growth inhibition, we used qPCR to quantify *M. genitalium* genomes in microbroth dilution assays. After assessing MIC endpoints by color change, we added 1/10 vol (20 µL) of Triton lysis solution (10% Triton X-100, 100 mM Tris HCl pH 8, 10 mM EDTA) to the wells and incubated the plates at 95°C for 15–30 min. Lysates were mixed by pipetting, diluted 1:10 in water, and then used directly for qPCR using a TaqMan assay that detects a portion of the 5′ region of *mgpB* (8, 42). Each PCR was performed in triplicate reactions, and then compared to a standard curve of known *M. genitalium* genomes prepared in quadruplicate. The drug concentration resulting in a 50% reduction (IC_50_) in *M. genitalium* genomes relative to untreated wells was calculated using a four-parameter logistic regression model. Genome quantities were determined in Vero cell cocultures using a similar method except that lysates were prepared using 45 µL of culture supernatant and 5 µL of Triton lysis solution.

### Whole-genome sequencing

Fusidic acid-resistant mutants were sequenced to identify the location of resistance-associated mutations. Resistant mutants were grown in 60 mm petri dishes in 4–5 mL SP-4 broth containing fusidic acid. When late log growth was observed the culture supernatant was discarded and the adherent cells were washed and scraped into phosphate-buffered saline (PBS). The cell suspension was centrifuged at 21,000 × *g* for 10 min and the cell pellet was resuspended in 150 µL of PBS. Total DNA was isolated utilizing the MasterPure Complete DNA and RNA purification kit (Lucigen, Middleton, WI), following the manufacturer’s instructions, with the exception that tubes were gently mixed by inversion instead of vertexing, to prevent DNA shearing and preserve high molecular weight DNA. Isolated DNA was suspended in nuclease-free water, quantified by Nanodrop, and prepared for sequencing using the Rapid Barcoding kit (Kit 14 chemistry, Oxford Nanopore Technologies, Oxford Science Park, UK). Libraries were sequenced using an R10.4.1 flow cell on the Mk1B MinION sequencing device according to the manufacturer’s instructions. Sequencing produced the following number of classified reads (N_50_ 4.0 kb): FAR1 328,234; FAR2 231,260; FAR3 346,834; FAR5 22,748; and FusR8 9,098. Read files were processed using SAMtools (43), aligned to the G37 reference genome using GraphMap (44), and then manually examined for mutations across the genome using the Integrative Genomics Viewer (45).

## Data Availability

Sequence data for the *Ramularia* sp. isolate were deposited in GenBank under accession no. PP476214.

## SUPPLEMENTAL MATERIAL

Supplemental figures aac.01006-24-s0001.docx NMR spectra.

ASM does not own the copyrights to Supplemental Material that may be linked to, or accessed through, an article. The authors have granted ASM a non-exclusive, world-wide license to publish the Supplemental Material files. Please contact the corresponding author directly for reuse.
