# Supplementary material for: The fungal natural product fusidic acid demonstrates potent activity against Mycoplasma genitalium
Source: Antimicrob Agents Chemother. 2024 Aug 29;68(10):e01006-24. doi: 10.1128/aac.01006-24 (PMC11459954; doi:10.1128/aac.01006-24)
Supplement: Supplemental figures — NMR spectra. [file aac.01006-24-s0001.docx]

**Supporting information figures**

**Fig. S1.** ^1^H NMR Spectrum of Fusidic acid (500 MHz, CDCl_3_)

**Fig. S2.** ^13^C NMR Spectrum of Fusidic acid (125 MHz, CDCl_3_)
